# Strengthening radon surveillance in British Columbia: Insights from a qualitative evaluation of the British Columbia Radon Data Repository

**DOI:** 10.14745/ccdr.v52i78a03

**Published:** 2026-07-30

**Authors:** Bridget Irwin, Jiayun Angela Yao, David McVea

**Affiliations:** 1 Canadian Field Epidemiology Program, Centre for Emergency Preparedness, Regulatory, Operations and Emergency Management Branch, Public Health Agency of Canada, Ottawa, ON; 2 Environmental Health Services, British Columbia Centre for Disease Control, Vancouver, BC; 3 School of Population and Public Health, University of British Columbia, Vancouver, BC

**Keywords:** radon, surveillance system, evaluation, qualitative

## Abstract

**Background:**

Radon is a carcinogenic gas that is the leading environmental cause of lung cancer in non-smokers. While its health risks are well-known, exposure to radon is variable and hard to determine, impeding risk assessment and mitigation. The British Columbia Centre for Disease Control (BCCDC) operates the British Columbia Radon Data Repository (BCRDR) to aggregate radon test results in support of research and public health action. This study evaluates the BCRDR, focusing on its usefulness and acceptability to those who contribute to and use the repository.

**Methods:**

Between November 2024 and July 2025, five contributors of radon data to the BCRDR and six users of radon data from the BCRDR were interviewed. In addition, six data holders, five data users, and 74 members of the public completed web surveys. Interviews and surveys were analyzed qualitatively using inductive thematic content analysis to generate themes.

**Results:**

Overall, users and contributors find the BCRDR useful and acceptable. Contributing data holders described the data submission process as quick and easy, and felt that the BCCDC addressed concerns. The repository supported a wide range of uses by a variety of stakeholders, most of whom found the data valuable. Respondents provided recommendations to further strengthen the repository.

**Conclusion:**

The findings affirm the BCRDR’s value for both those contributing data and those using it. Responses can inform enhancements to the existing provincial repository and guide the development of future radon surveillance systems as well.

## Introduction

Radon is a carcinogenic gas formed during the natural breakdown of uranium in soil and rock. Radon poses minimal risk outdoors, where it quickly dilutes (1), but it can accumulate to high levels indoors. Globally, prolonged exposure is the leading cause of lung cancer among non-smokers, contributing to over 3,000 deaths each year in Canada alone (1,2). To reduce this risk, Health Canada recommends home remediation when average indoor radon concentrations exceed 200 Bq/m^3^ (3). Nevertheless, exposure to any level of indoor radon increases the risk of lung cancer (3).

Radon enters buildings through cracks and other openings, and indoor concentrations vary significantly according to the characteristics of the geography and built environment. Radon levels are highest in regions with elevated uranium levels, including northern and interior British Columbia (BC) (3). However, even within neighbourhoods of similar geology, indoor radon levels can differ between buildings because of design, age, ventilation, and maintenance (4,5). Given these factors, and because it is colourless and odourless, the only way to know if a building has elevated radon is to test for it, either using a self-test kit or hiring a certified radon measurement professional (3). In Canada, testing and remediation are largely self-driven and hindered by a lack of awareness: only 58% of households had heard of radon in 2023, and just 12% of non-apartment households that were aware of the gas had tested for it (6).

While the health effects of radon are well-documented, assessing risk can be difficult due to variability in exposure and limited testing. Although many countries maintain national radon databases, most rely on cross-sectional survey data and are not designed to function as centralized, continuously updated surveillance repositories (7). In Canada, radon surveillance is not standardized, and the available measurement data have historically been disjointed and difficult to access, held separately by the various entities that have conducted testing. The lack of a comprehensive system has made it difficult to characterize radon exposure across the country and has hindered action and mitigation.

To address this for the Province of BC, the BC Centre for Disease Control (BCCDC) launched the BC Radon Data Repository (BCRDR) in 2021. The BCRDR and its development have been described elsewhere (8). The repository aggregates indoor radon measurements from volunteer data holders into a single system for research, risk assessment, and action. The data are cleaned and anonymized according to a data sharing agreement (DSA) and are available to researchers on request. Repository data are also displayed in the public-facing BC Radon Map, which maps summary statistics at different geographies. As of July 2025, the BCRDR contained 45,060 total measurements from over 23,000 presumed unique buildings.

As the first of its kind in Canada, the BCRDR represents an important advancement in radon surveillance. However, there is an opportunity to further improve the function and utility of the repository, particularly as calls grow for a similar, national level radon surveillance system. This study evaluates the experiences of those who contribute to and use the BCRDR. The objectives were to 1) understand the acceptability of the BCRDR from the perspective of data contributors; and 2) describe how stakeholders use the BCRDR and identify areas for improvement. The findings will inform enhancements to the existing provincial repository and may guide the development of radon data repositories in other jurisdictions, both in Canada and internationally.

## Methods

### Study design

To formally evaluate the BCRDR, this qualitative study adapted methods from the European Centre for Disease Prevention and Control’s *Data quality monitoring and surveillance system evaluation* handbook (9) and the United States Centers for Disease Control and Prevention’s *Updated Guidelines for Evaluating Public Health Surveillance Systems* (10). Informed by the study objectives, two surveillance system performance attributes were addressed: acceptability and usefulness. A summary of these attributes is presented in **Table 1**.

**Table 1:** Attributes assessed in the British Columbia Radon Data Repository surveillance system evaluation

Attribute	Definition in the context of the BCRDR	Methods	Analysis/synthesis
Acceptability	The willingness of radon data providers to participate in the BCRDR surveillance system, now and in the future.	Survey of data holders	Qualitative thematic content analysis of open-text survey fields
		Semi-structured interviews with data holders	Qualitative thematic content analysis of interview transcriptions
Usefulness	The usefulness of the BCRDR and the associated British Columbia Radon Map for informing public health action and contributing to the prevention and control of adverse health outcomes related to radon exposure.	Survey of data users	Qualitative thematic content analysis of open-text survey fields
		Semi-structured interviews with data users	Qualitative thematic content analysis of interview transcriptions
		Public web survey	Qualitative thematic content analysis of open-text survey fields

Abbreviation: BCRDR, British Columbia Radon Data Repository

### Study participants

Three relevant stakeholder groups were identified:

1) Radon data holders, including all individuals and organizations collecting indoor radon data in BC, regardless of whether they contribute these data to the BCRDR

2) British Columbia Radon Data Repository users, including researchers and public health practitioners who use the BCRDR data tables/BC Radon Map in their work

3) Members of the public, who access the BC Radon Map

Categories were not mutually exclusive, and several contributors were also users of the BCRDR.

### Data collection

To inform the evaluation, three web-based surveys were developed using Checkbox (summarized in **Table 2** and presented in **Appendix**, Supplemental material), each tailored to a different stakeholder group. The data holders survey assessed experiences contributing to the BCRDR and was distributed to all 41 known radon data holders across the province between May and August 2025. The data users survey examined how the BCRDR and BC Radon Map are used in practice and was distributed between May and August 2025 to stakeholders at 17 relevant organizations, including local and provincial public health and provincial ministries of health, housing, and education. The public survey gathered feedback from users of the BC Radon Map on its usefulness for increasing knowledge and assessing risk. It was promoted on the map website between November 2024 and April 2025, and participants could enter a draw for a $100 gift card as an incentive. All surveys were pilot tested prior to launch.

**Table 2:** Summary of the surveys used to assess the acceptability and usefulness of the British Columbia Radon Data Repository

Audience	Description	Date	Distribution	Responses
Data holders	The data holders survey sought to understand the experiences of radon data holders contributing data to the BCRDR. Questions covered four domains: Background information (e.g., tests conducted per year, information collected) Acceptability of participation (e.g., concerns about participation, time and resources required to participate) Reporting and dissemination (e.g., frequency of reporting, awareness of BCRDR/BC Radon Map uses) General feedback (e.g., challenges, suggestions for improvements)	Launched May 2025. Results extracted August 7, 2025.	Distributed by email to all 41 known radon data holders from across the province, including 12 current BCRDR contributors and 29 data holders who have never participated in the repository.	Eight survey responses received, including six from current/former contributors and two from data holders who have never participated. An additional five interviews with data holders were completed, for an overall response rate of 31.7%.
Data users	The data users survey aimed to explore how the BCRDR and BC Radon Map are used in practice and to collect feedback on their functionality. Questions covered five domains: Background information (e.g., role/organization, ways of using the data) Satisfaction with the various elements of the data/map (e.g., timeliness, accessibility, ease of interpretation) Usefulness of the data/map (e.g., changes to policy/practice resulting from the data/map, elements/variables missing) Acceptability of using the data/map to inform work (e.g., comfort level, concerns) General feedback (e.g., challenges, suggestions for improvement)	Launched May 2025. Results extracted August 7, 2025.	Distributed by email to stakeholders at 17 organizations believed to be using the data, including local and provincial public health and the ministries of health, housing, and education.	Four responses received, including two from users of the map, one from a user of the data tables, and one from a user of both the map and tables. An additional six interviews with data users from five organizations were completed, for an overall response rate of 52.9%.
Public	The public web survey was intended to gather feedback from the general public on the BC Radon Map, specifically on its usefulness for assessing risk. Questions covered four domains: Background information (e.g., role, age, FSA of residence) Radon knowledge before and after using the map (e.g., of health risks, of spatial distribution) Usefulness of the map (e.g., usefulness for assessing risk, functionality of the map, intended actions) General feedback (e.g., challenges, suggestions for improvements) A chance to win a $100 gift card was offered as an incentive to encourage participation	Launched on the BC Radon Map webpage November 2024 (to coincide with Radon Action Month). Remained active until April 2025. Results extracted April 22, 2025.	Link to survey was displayed at the top of the map to capture general users.	Seventy-four responses received.

Abbreviations: BC, British Columbia; BCRDR, British Columbia Radon Data Repository; FSA, Forward Sortation Area

To supplement the information collected from the surveys, interviews were also conducted with a subset of key informants, prioritizing the data holders who contribute the most measurements and the users who formally requested BCRDR data. Interviews lasted 30–90 minutes in duration and took place virtually via Microsoft Teams or Zoom using a semi-structed interview guide (Appendix, Supplemental material). Interview questions were adapted from the surveys and were open-ended, designed to elicit more detailed feedback. With the permission of the interviewees, all interviews were recorded and transcribed. This study is considered a program evaluation and was thus exempt from research ethics board approval.

### Data analysis and synthesis

Guided by Braun and Clarke’s framework (11), inductive thematic content analysis was used to identify key themes emerging from the survey and interview data by stakeholder group and surveillance attribute. One author conducted the primary analysis, reading interview transcripts multiple times and noting trends, inconsistencies, and contraindications across the data. The author then coded the transcripts and generated themes using a latent approach and then discussed the codes and themes with the broader research team to ensure clarity, consensus, and consistency with the data. Finally, themes were refined and supporting quotations were selected from interviews to represent each theme.

### Findings

Results are organized according to the themes derived from the qualitative analysis in relation to each surveillance attribute. Participant details are summarized in **Table 3** and **Table 4**, while recommendations based on the findings are presented in **Table 5**.

**Table 3:** Participant details

Participant number	Data collection type	Relationship to BCRDR
P1	Interview	Current data provider and user of the BCRDR data and BC Radon Map
P2–P4	Interview	Current data provider and user of the BC Radon Map
P5	Interview	Current data provider and user of the BCRDR data
P6, P9, P10	Interview	User of the BCRDR data
P7, P8	Survey	User of the BC Radon Map
P11, P12, P16, P17	Survey	Previous data provider
P13, P15	Survey	Data holder that has never contributed data
P14, P18	Survey	Current data provider
P19–P92	Survey	Public user of the BC Radon Map

Abbreviations: BC, British Columbia; BCRDR, British Columbia Radon Data Repository

**Table 4:** Participants by role/organization

Role/organization	Number of participants (n=92)
Academic institution	4 (4.3%)
Federal government	3 (3.3%)
Local government	1 (1.1%)
Member of the public	42 (45.7%)
Non-governmental radon organization	7 (7.6%)
Provincial government	1 (1.1%)
Public health practitioner	16 (17.4%)
Realtor	2 (2.2%)
Regional public health authority	4 (4.3%)
Researcher	5 (5.4%)
Other	5 (5.4%)
Prefer not to say	3 (3.3%)

**Table 5:** Summary of recommendations resulting from the British Columbia Radon Data Repository evaluation

Recommendation	Specific actions
Strengthen trust, reciprocity, and engagement with data holders to maximize the acceptability of participation in the BCRDR	• Continue emphasizing the privacy protections and anonymization processes outlined in the data sharing agreement during recruitment. • Follow up with non-contributing data holders to address outstanding concerns. • Improve reciprocity by providing periodic updates to contributors on how the BCRDR data are being used.
Maintain the current low-burden data submission process to ensure participation in the BCRDR is easy for contributors	• Maintain the simple spreadsheet-based data submission process and the current annual submission frequency, which aligns well with radon testing cycles. • Continue to provide onboarding guidance and support for new contributors. • Review internal processes related to data sharing agreements to minimize onboarding delays. • Where feasible, provide minor support to further reduce workload (e.g., templates, gentle reminders during data submission periods).
Enhance awareness of the BCRDR and BC Radon Map to encourage usefulness	• Continue to promote the BCRDR and BC Radon Map as a trusted, authoritative public resource. • Strengthen outreach to ensure the existence and availability of the BCRDR and BC Radon Map are widely known.
Improve the clarity, interpretation, and risk communication of the BC Radon Map for lay audiences	• Continue to refine the messaging on the BC Radon Map to emphasize that all homes should be tested regardless of map colour. • Add clearer explanations and caveats about the limitations of radon mapping. • Develop a short tutorial embedded directly in the map to explain the various features and functions. • Add clearer action-oriented messaging to the map (e.g., “you should test your home regardless of the map result”). • Add plain-language definitions of the map indicators (e.g., 95^th^ percentile, different geographic levels).
Increase the transparency and usefulness of the BCRDR and BC Radon Map through increased context and granularity	• Improve transparency around sample sizes and uncertainty (e.g., warnings when data are sparse). • Encourage more data collection in under-tested regions to improve the spatial resolution of the data. • Explore opportunities to incorporate additional variables, contextual information, and filters wherever possible (e.g., filters for building type, mitigation system, integration of non-residential testing data, information on geology, lung cancer rates, local testing resources, etc. in the map pop-up boxes).

Abbreviations: BC, British Columbia; BCRDR, British Columbia Radon Data Repository

### Acceptability

Five current BCRDR contributors were interviewed, while an additional eight completed surveys (six current/former contributors and two data holders who have never contributed). From the coding framework, four themes relating to acceptability were generated.

#### Data holders were willing to participate, despite initial concerns

Most current contributors were willing to participate in the BCRDR and would choose to do so again if given the chance. While respondents recalled some initial concerns surrounding privacy and confidentiality, these were largely resolved through discussions with the BCCDC and a DSA outlining data anonymization and ownership principles. One contributor noted that “confidentiality is important to us, so we don’t work with everybody, but I think between the way the data was being shared with the public and then the confidentiality agreement that we were able to sign with BCCDC, we were good to share the data to the point that we are sharing it” (P3). Among the two data holders who have never contributed, both cited privacy concerns, but were open to further discussions with the BCCDC.

#### Onboarding and data submission were generally straightforward

Contributors generally reported no issues onboarding to the BCRDR and praised BCCDC staff for their support, with one sharing “we had a person who [would] just tell us everything we need[ed] to know…so it was actually super easy” (P1). One contributor described an 18-month delay in signing the DSA, referring to the experience as “painful” (P2). However, they acknowledged that this occurred during the COVID-19 pandemic and was not typical. Overall, contributors appreciated the simplicity of the data submission process, with data sent in a spreadsheet via email. One disclosed that “I work with a variety of other groups with whom I need to share data, and sometimes I need to try to upload it to their portal or something, and that’s a nightmare” (P3).

#### Participation in the British Columbia Radon Data Repository did not pose a significant burden

Time spent on repository-related activities, including extracting, cleaning, and submitting the data, was minimal, although a handful of contributors reported that it wasn’t a priority for them, and so could be challenging. As one respondent put it, “finding the time to actually get those two hours, that’s the hard part. But the actual labour, once I find the time to do it, is not that bad” (P5). Annual data submission was considered appropriate given the long-term nature of radon testing, and one respondent noted that “radon testing is an annual thing really…a year is about right” (P5).

#### Perceived value of contributions was mixed

Knowledge of how BCRDR data are being used in practice varied among contributors. While all were aware of the BC Radon Map, there was more uncertainty surrounding the data tables, and many did not know the specifics of their usage, leading them to question the value of their participation. One provider commented “I can see it being a problem if nobody’s requesting it…what are we holding this for?” (P1). Some contributors expressed that receiving results and deliverables from the BCRDR may increase their motivation to share, with one stating that “it would be nice to know…if I knew that something was happening with [the data], it might help” (P2). Others were less concerned about the uses of the data, and one explained “I have my own data…I’m not generally looking for other [data]” (P3).

## Results

### Usefulness

Six users of the BCRDR data tables and/or BC Radon Map were interviewed and 78 surveys were received (four from professional users of the BCRDR data tables/BC Radon Map and 74 from members of the public). From the coding framework, six themes relating to usefulness were generated.

#### The British Columbia Radon Data Repository data and British Columbia Radon Map supported diverse uses

British Columbia Radon Data Repository users mainly used the data tables for research, while the map supported a broader range of purposes, such as identifying under-tested areas, informing interventions, and driving policy changes. Indeed, many stakeholders felt that the BCRDR had contributed to recent BC building code amendments, which require all new builds to install radon rough-ins, with one affirming that “the work done through this map…has enabled the BC building code to…no longer have different zones” (P2). Users also described the map as a trusted source to point to when engaging with communities and external partners. As one user said, “the fact that it’s from the BCCDC, it is a powerful tool” (P4).

While all stakeholders found the BCRDR useful, opinions on its importance varied. Some described having a single source of data for the whole province as essential, whereas others saw it as more supplementary, noting that “it’s interesting [but] I wouldn’t say it plays a pivotal role” (P3). Among public users, feedback was overwhelmingly positive and 90% of respondents rated the map as very useful for assessing their likelihood of living in a high radon home. Most reported increased knowledge of the health effects (70.3%) and spatial distribution (87.8%) of radon after viewing the map, and more than half intended to get their home tested.

#### Some users had concerns about radon mapping

Most users felt comfortable using the BC Radon Map in their work, with one stating that “it’s a very robust radon map by global standards now” (P1). Despite this, many noted the limitations of radon mapping in general, such as masking local variation and oversimplifying risk. As one user observed, “peoples’ tendency seems to be look at a map, see ‘oh, it’s not that bad in my region, therefore I don’t need to test’, when the reality of radon is even if a very small number of homes in your region have elevated radon, there’s no way of telling if you’re one of them” (P3). Differences in radon exposure likelihood by building type were also downplayed in the map, with users noting “if [a person] lives in a 17-story apartment building… they probably have negligible risk. If [a] person [is] in a single-family detached house…their risk is an order of magnitude higher, but they look at the same map and it’s [low risk] for both of them” (P5). Users acknowledged the BCCDC’s efforts to mitigate these issues and felt the map had addressed them to the extent possible through design choices (e.g., colours) and messaging.

#### Interpretation could be challenging for lay users

Professional users of the BC Radon Map found it easy to interpret after an initial learning period, but many felt it could confuse lay users. One user remarked that “I’ve heard [BCCDC staff] explain some of the…different ways you could display the data, and I thought, ‘oh, that’s really interesting, but I don’t think your average person is going to interpret it that way’” (P3). Public feedback confirmed this, and several respondents were unsure whether they should test their homes for radon based on the information in the map. For example, one respondent who lived in an area with very few homes above the threshold felt that the map offered little practical value since the recommendation was to test regardless. Users from all groups suggested that a tutorial be developed and incorporated directly into the map.

#### Granularity was insufficient for some users

Opinions on the granularity of the data varied by user location, with those in regions with smaller sample sizes reporting limited utility. Several users noted that it’s hard to make a generalized statement to assess risk and public respondents, in particular, requested finer geographic detail beyond municipalities (the smallest level currently available).

#### Additional information would enhance usefulness

British Columbia Radon Data Repository users of all types expressed a desire for additional information, such as more filters and contextual data. Although data holders rarely collect these details at present, which makes implementation difficult in practice, suggested future additions, if the data become available, include filters for dwelling type, presence or absence of a radon mitigation system, and whether the test occurred before or after remediation. One user remarked that “if you had data showing people with rough ins…or conversely, people who’ve had full mitigation by a certified mitigator…that would be awesome” (P1). Users also requested that measurements from workplaces, schools, and daycares be included on the map to increase the number of data points. They also asked for additional contextual information in the pop-up box when a region of the map is selected, such as geologic radon concentrations, lung cancer rates, and local testing resources.

## Discussion

This evaluation examined the acceptability and usefulness of the BCRDR, two attributes critical to surveillance system sustainability and impact (8,9). To the authors’ knowledge, this study is the first formal evaluation of an integrated radon surveillance database to date.

Overall, respondents viewed the repository positively but identified some opportunities for improvement. Acceptability was high among data providers, who described onboarding and data submission as quick and easy, requiring minimal additional time or resources. Concerns about privacy and confidentiality, while pervasive, were largely resolved through the DSA, and no other major challenges were reported. Some providers requested greater reciprocity, however, noting that they rarely received feedback on how their data are used. This created some uncertainty about the value of continued participation, and enhanced communication may therefore improve motivation and strengthen trust between data providers and the BCCDC.

Usefulness was also rated highly, and respondents from different stakeholder groups used the BCRDR for diverse applications, such as assessing the likelihood of living in a high radon home, guiding research, and informing program planning and policy. While most users found the information valuable, some areas for improvement emerged, largely centred around the BC Radon Map. Interpretation was identified as a challenge for lay users in particular, and the potential harms of misinterpretation, a concern frequently raised in radon mapping literature (12), were highlighted. Many users identified the inclusion of additional data as key in reducing misinterpretation, as well as in improving the overall utility of the BCRDR, and called for contextual information (e.g., geologic features, lung cancer rates), measurements from non-residential buildings (e.g., daycares, workplaces) and expanded data filters (e.g., dwelling type, mitigation status), along with a map tutorial.

The themes identified in this evaluation can be used both to improve BC’s existing provincial radon surveillance system and to guide the development of similar systems, ensuring that acceptability and usefulness remain central to their design. Specifically, the findings underscore the importance of a streamlined data submission process to minimize the burden of reporting on contributors, as well as the need for transparent data governance principles and open and ongoing communication. They also highlight the value of incorporating other relevant information wherever possible to maximize usefulness across stakeholder groups, and the need to design maps and other data products with a lay audience in mind, with priority given to simplicity and ease of interpretation to reduce the risk of misinterpretation. Such lessons will be particularly important as the BCCDC continues to support other provinces and territories across Canada in aggregating and mapping their own radon data holdings, with the goal of eventually creating an integrated national repository based on the BCRDR. They may also inform the enhancement of international radon surveillance programs, many of which have highlighted similar challenges related to data integration, accessibility, and usability (13–16).

### Limitations

This study had a few limitations. First, response rates were low among both radon data holders (31.7%) and users (52.9%), resulting in potential bias. Indeed, respondents who provided feedback may have been more engaged with the BCRDR than those who did not, and more likely to view it favourably as a result. This is particularly important when assessing the acceptability of the surveillance system, as limited feedback was received from data holders not currently contributing to the repository, who may hold systematically different perspectives. Similarly, the public survey recruited participants directly through the map website and therefore likely over-represents individuals who are already aware of radon and its health risks compared to the general population. The findings may therefore overestimate the acceptability and perceived usefulness of the BCRDR.

## Conclusion

The BCRDR is generally acceptable and useful to stakeholders. Insights from this evaluation can guide enhancements to the provincial system and inform future national-level radon surveillance, both in Canada and abroad.

## Appendix

Supplemental material is available upon request to the author: bridget.irwin@bccdc.ca
